# Associations of Sedentary Behavior and Screen Time with Human Gut Microbiome Composition and Diversity

**DOI:** 10.3390/life14030363

**Published:** 2024-03-09

**Authors:** Maximilian T. Antush, Onesmo B. Balemba, Sarah A. Hendricks, Morgan Flynn, Rayme Geidl, Chantal A. Vella

**Affiliations:** 1 Department of Movement Sciences, University of Idaho, Moscow, ID 83843, USA; antushm@uidaho.edu (M.T.A.);; 2 Department of Biological Sciences, University of Idaho, Moscow, ID 83843, USA; obalemba@uidaho.edu; 3 Washington, Wyoming, Alaska, Montana, and Idaho Medical Education Program, University of Idaho, Moscow, ID 83843, USA; 4 Institute for Interdisciplinary Data Sciences, University of Idaho, Moscow, ID 83843, USA

**Keywords:** sedentary behavior, screen time, fecal microbiota, gut microbiota, insulin resistance, prediabetes, type 2 diabetes

## Abstract

Human gut microbiome richness, diversity, and composition are associated with physical activity and impaired glycemic control; however, the associations with sedentary behavior and screen time are not as well-established. This study evaluated associations of sedentary behavior and screen time with the alpha diversity and composition of the human gut microbiome in adults with and without impaired glycemic control. Sedentary behavior and screen time data were collected via survey from 47 adults (38% with impaired glycemic control). Microbiome composition and alpha diversity were determined in fecal microbial DNA. Sedentary behavior was negatively associated with the number of observed operational taxonomic units (OTUs), Chao 1 Index, and Fisher’s Alpha Index. These associations were slightly attenuated but remained significant when controlling for covariates. Screen time was negatively associated with the number of observed OTUs, Shannon Index, and Fisher’s Alpha Index; however, only the association with observed OTUs was independent of all covariates. Our findings suggest sedentary behavior and screen time may be significant influencers of compositional changes in human gut microbiota. This may be a potential mechanism linking sedentary behavior and screen time to an increased risk of type 2 diabetes.

## 1. Introduction

Sedentary behavior, defined as any waking behavior in a seated, reclined, or lying position expending ≤ 1.5 metabolic equivalents, is associated with an increased incidence of type 2 diabetes (T2D) [1,2,3]. Adults in the United States (US) spend approximately 40 to 50% of their waking hours, an average of 6 to 8 h per day, engaged in sedentary behaviors, with prevalence greater for older adults [3]. Accumulating evidence suggests high levels of sedentary behavior are associated with an increased risk for T2D [1,3]. Yet the mechanisms linking sedentary behavior to increased risk of T2D remain unclear. Given that approximately 96 million adults in the US have prediabetes and over 37 million have diabetes, the link between sedentary behavior and T2D warrants further investigation [4].

The effects of sedentary behavior on chronic disease risk, including T2D, have been shown to be independent of physical activity levels [5,6,7]. Specifically, a recent meta-analysis that included nine studies and over 400,000 participants indicated high levels of sitting time were associated with an increased incidence of T2D after adjusting for physical activity [1]. Additionally, previous studies have reported screen time, a type of sedentary behavior that involves using a device with a screen such as a smart phone, computer, or television, is associated with impaired glycemic control in adults [8,9]. Indeed, Dunstan et al. (2004) reported that the odds of abnormal glucose metabolism were higher in Australian men (OR 1.16) and women (OR 1.49) who watched television more than 14 h per week compared to those who watched 7 or fewer hours per week [8].

Compositional changes in gut microbiota have been associated with impaired glycemic control and T2D [10,11,12]. Larsen et al. (2010) reported *Firmicutes*/*Bacteroidetes* (F/B) ratios were significantly correlated with plasma glucose during an oral glucose tolerance test, but not body mass index (BMI), in a sample of adult males with 50% of participants diagnosed with T2D [10]. Le Chatelier et al. (2013) reported that individuals with low gut microbial species richness, an indicator of alpha (α)-diversity, had increased insulin resistance compared to those with high gut microbial species richness [11]. Similarly, Zhang et al. (2013) reported that gut microbiota α-diversity was significantly associated with fasting plasma glucose across a sample that included adults with normal glucose tolerance, prediabetes, and T2D [12]. However, a 2020 review reported that while several studies have found a correlation between T2D and gut microbiota α-diversity and F/B ratios, other studies reported no correlation [13].

A growing number of studies have reported associations between higher levels of physical activity and gut microbiome compositions that are linked to positive health outcomes [14,15,16,17]. Specifically, Clarke et al. (2014) found that professional rugby athletes had significantly greater gut microbiota α-diversity compared to sedentary controls [15]. To our knowledge, few studies have attempted to describe the effects of sedentary behavior on gut microbiota composition [18,19,20], and only two studies have assessed the influence of screen time [21,22]. Compositional changes in the human gut microbiota may be a potential mechanism that links sedentary behavior and screen time to the incidence of T2D. Therefore, the purpose of this study was to evaluate associations of sedentary behavior and screen time with human gut microbiota α-diversity and F/B ratio in adults with and without impaired glycemic control. We hypothesized that sedentary behavior and screen time would be inversely associated with α-diversity and positively associated with F/B ratio, and that these associations would be independent of relevant covariates. This study is part of a larger study investigating lifestyle and gut microbiome influence on the development of T2D and complications of T2D entitled, “A study to explore novel causes of diabetes neuropathy and dysmotility in the gut”, which was funded by NIH, NIDDK Diabetic Complications Consortium.

## 2. Materials and Methods

### 2.1. Participants

A convenience sample of 47 adults aged 18 years and older, with and without type 2 diabetes, was recruited from a university and its surrounding community through email announcements, newspaper advertisements, and flyers. All participants completed a pre-screening questionnaire over the phone with a trained researcher to determine eligibility for the study. Participants were excluded if they reported a history of any of the following: gastric bypass surgery, irritable bowel syndrome, Crohn’s disease, colitis, colon cancer, celiac disease, multiple sclerosis, Parkinson’s disease, Alzheimer’s disease, type 1 diabetes, or current pregnancy. Participants who were enrolled in the study completed two laboratory visits within eight days and completed a diet history questionnaire between visits. The study was approved (27 July 2020) by the University of Idaho Institutional Review Board (protocol 20-098) and all participants provided written informed consent. The study was conducted in accordance with the Declaration of Helsinki for experiments involving human subjects.

### 2.2. Subject Demographics and Health History

A self-reported health history questionnaire was used to assess demographics (age, sex, race and ethnicity, and marital status), medical history, family health history, current medications and supplements, smoking and/or tobacco use, exercise participation, and menstrual history. Body mass was recorded to the nearest 0.01 kg and height was measured as the average of two measurements, to the nearest 0.1 cm, using a calibrated digital scale with a stadiometer (Seca 220; Seca, Hamburg, Germany). Body mass index (BMI) was calculated as body mass in kilograms divided by height in meters squared.

### 2.3. Sedentary Behavior, Screen Time, and Physical Activity

Self-reported sedentary behavior was collected using the Sedentary Behavior Questionnaire (SBQ). The SBQ has been demonstrated to have acceptable 2-week test–retest reliability and validity compared to accelerometer-measured inactivity and sitting time from the International Physical Activity Questionnaire (IPAQ) in normal weight and overweight adults [23]. The first page of the SBQ prompts respondents to report the average time per day during their waking hours, on a typical weekday, spent engaged in nine different sedentary behaviors: watching television or streaming services, playing computer or video games, listening to music, talking or texting on a phone, doing paperwork or computer work, reading a book or magazine, playing a musical instrument, doing artwork or crafts, and driving or riding in a vehicle. The second page of the SBQ uses the same prompt and sedentary behaviors but asks respondents to report on a typical weekend day.

For each of the nine sedentary behavior categories, response options include none, 15 min or less, 30 min, 1 h, 2 h, 3 h, 4 h, 5 h, and 6 h or more. Time spent in each of the nine sedentary behavior categories was converted to hours, summed, and then multiplied by the number of days per week for each prompt (5 days for weekdays and 2 days for weekend days). Total sedentary time was grouped into three domains—leisure sedentary time (watching television or streaming services, playing computer or video games, listening to music, talking or texting on a phone, reading a book or magazine, playing a musical instrument, and doing artwork or crafts), occupational sedentary time (doing paperwork or computer work), and passive transportation (driving or riding in a vehicle).

Self-reported screen time was obtained using the Screen-time Questionnaire (STQ). The STQ has been demonstrated to have acceptable 3-day test–retest reliability [24]. The STQ asks respondents to report the total amount of time spent using five different types of screen-based devices (television, TV-connected devices, computer, smartphone, and tablet) as their primary activity, in hours and minutes per day. The responses are divided into screen use during an average weekday, an average weeknight, and an average weekend day. Responses to each question, for weekday and weekend day categories only, were converted to minutes, summed, and then multiplied by the number of days per week (5 days for weekdays and 2 days for weekend days). The weekday and weekend totals were summed and divided by seven to get average screen time per day.

Self-reported moderate-to-vigorous physical activity (MVPA) was assessed using the long-form IPAQ. The IPAQ has been demonstrated to have acceptable 3- to 7-day test–retest reliability and validity compared to accelerometer-measured MVPA [25]. The IPAQ is designed to assess moderate- and vigorous-intensity physical activity that respondents have engaged in during the previous 7 days, for at least 10 min at a time, across four different domains. The domains covered by the IPAQ include “job-related physical activity”, “transportation physical activity”, “housework, house maintenance, and caring for family”, and “recreation, sport, and leisure-time physical activity”. For each question on the survey, participants responded with the number of days they engaged in that activity and the average amount of time per day spent on the activity. Average MVPA was calculated by multiplying the number of days, total minutes, and task-specific metabolic equivalent (MET), and this was expressed as MET-min per week.

### 2.4. Measurement of Glucose, Lipids, and Hemoglobin A1c

Two fingerstick samples of blood were collected to measure glucose, high-density lipoprotein (HDL) and low-density lipoprotein (LDL) cholesterol, total cholesterol, triglycerides, and hemoglobin A1c (HbA1c). The participant’s finger was warmed, cleaned with 70% isopropyl alcohol, and allowed to air-dry prior to collection. A disposable safety lancet (SurgiLance; MediPurpose, Duluth, GA, USA) was used to puncture the middle or ring finger of the non-dominant hand and the first droplet of blood was wiped clean. The first sample (~40 µL) was drawn into a lithium heparin capillary tube, transferred into the reagent cartridge, and analyzed immediately using the Cholestech LDX Analyzer (Abbott Point of Care Diagnostics, Princeton, NJ, USA) under standardized operating procedures to obtain blood glucose and lipids. The second sample (~1 µL) was drawn from the same puncture site into a capillary tube, inserted into the reagent cartridge, and analyzed immediately using the DCA Vantage analyzer (Siemens Healthcare Diagnostics, Tarrytown, NY, USA) under standardized operating procedures to obtain HbA1c [26].

### 2.5. Diet History Questionnaire

Between laboratory visits, participants completed the Diet History Questionnaire III (DHQ III) to collect dietary intake data. The DHQ III is a validated web-based food frequency questionnaire developed by the National Cancer Institute Division of Cancer Control and Population Sciences based on 24 h dietary recall data from the National Health and Nutrition Examination Surveys conducted from 2007 to 2014 [27,28,29]. The DHQ III prompts respondents to report food intake over the last month and consists of 135 food and beverage line items and 26 dietary supplement questions that lead to a total 263 possible selections. The questionnaire summary quantifies the average daily intake of macro- and micronutrients.

### 2.6. Stool Sample Collection, Taxonomic Identification, and Alpha Diversity Analysis

Participants were provided a stool sample collection kit and detailed verbal and written instructions for the collection and storage of the sample. All samples were stored on ice in a Styrofoam cooler and returned to the laboratory within 24 h of collection. The samples were frozen at −80 °C prior to extraction. The average number of days between the initial laboratory visit and collection of the fecal sample was 4.8 ± 2.1 days.

Total genomic DNA was extracted from the fecal samples using the QIAamp PowerFecal Pro DNA kits (Qiagen, Hilden, Germany) and the Wave StrainID Kits (Intus Biosciences, Farmington, CT, USA), and SetA and SetZ were used to produce amplicons that spanned the full-length 16S, ITS, and partial 23S rRNA genes [30]. These amplicons were used to generate sequencing libraries using PacBio SMRTbell Express Template Prep Kit v.3.0 (Pacific Biosciences, Menlo Park, CA, USA) and SBanalyzer v.3.0 (Intus Biosciences) was used to demultiplex and assign taxonomic identification to all reads by mapping to the Athena database [30,31]. The sequences in this analysis were clustered by similarity using a threshold of 97% and put into bins called “Operational Taxonomic Units” (OTUs) [30]. Microbiome composition was expressed as the F/B ratio. Alpha diversity was expressed as observed OTUs, Shannon Index, Chao 1 Index, and Fisher’s Alpha Index.

### 2.7. Covariates

Body mass index, MVPA, glucose, HDL cholesterol, and triglycerides were used as covariates in the analyses, as these variables were significantly correlated to the dependent variables. Participant age, sex, HbA1c, low-density lipoprotein LDL cholesterol, total cholesterol, and dietary intake were not significantly correlated with any of the dependent variables; therefore, they were not included as covariates in the analyses.

### 2.8. Sensitivity Power Analysis

We performed a post-hoc sensitivity power analysis using GPower (Version 3.1.9.2, Universität Kiel, Kiel, Germany) for linear multiple regression. Given our sample size of 47 and the 6 predictors in our final linear regression model, our designed study showed a 90% power to detect effects (f^2^) of at least 0.433 [32].

### 2.9. Statistical Analysis

All data were examined for normal distribution using the Shapiro–Wilk test for normality. Data that were non-normally distributed were transformed and rechecked for normality. Characteristics of the sample were summarized with mean and standard deviation (SD) for normally distributed continuous variables, median and interquartile range for non-normally distributed continuous variables, and frequency and percentage of the study sample for categorical variables. Simple correlations were used to assess associations between variables. Multivariable linear regression analyses were used to determine associations of domain-specific sedentary behavior and device-specific screen time with the F/B ratio and measures of α-diversity. The initial model (model 1) was unadjusted. Model 2 was adjusted for MVPA and BMI. Model 3 included Model 2, as well as adjustments for glucose, HDL cholesterol, and triglycerides. All data analyses were conducted with IBM SPSS^®^ v25 (IBM, Armonk, NY, USA), and α = 0.05 was used to determine statistical significance.

## 3. Results

### 3.1. Participant Characteristics

**Figure 1:**
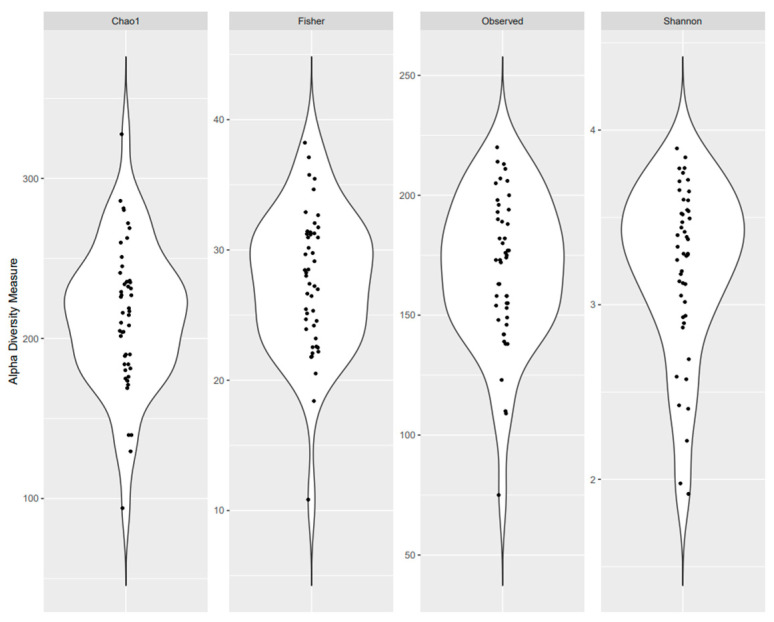
Microbial alpha diversity as measured by the Chao 1 Index, Fisher’s Alpha Index, observed OTUs, and Shannon Index of fecal samples.

The study participants’ characteristics are presented in Table 1. Overall, the mean age of participants was 51.0 years; 61.7% were women, 78.7% were non-Hispanic white, and 93.6% were non-smokers. On average, participants were classified as overweight with a mean BMI of 29.6 kg/m^2^ and 38.3% had prediabetes or T2D, while 46.8% had a family history of T2D. The average total time spent engaged in sedentary behavior was 7.8 h per day and the average total screen time was 7.0 h per day. Distributions of α-diversity measures are presented in Figure 1.

### 3.2. Taxonomic Identification

**Figure 2:**
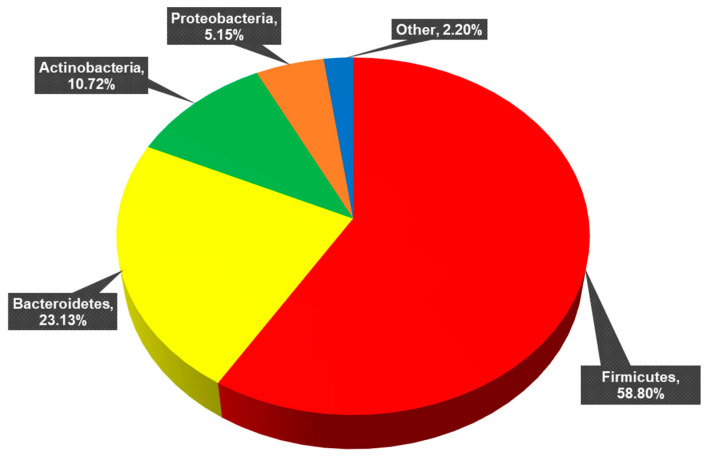
Percentage of each phylum present across all samples. *Firmicutes* (mean percent total reads across samples: 58.80 ± 16.85%), *Bacteroidetes* (23.13 ± 15.90%), *Actinobacteria* (10.72 ± 10.37%), *Proteobacteria* (5.15 ± 5.76%). Other: *Verrucomicrobia* (1.40 ± 2.99%), *Tenericutes* (0.61 ± 1.71%), *Elusimicrobia* (0.03 ± 0.22%), *Synergistetes* (0.02 ± 0.08%), *Fusobacteria* (0.02 ± 0.07%), *Lentisphaerae* (0.002 ± 0.007%), and *Cyanobacteria* (0.0006 ± 0.0044%).

Full taxonomic identification is reported in Section 3.1 of Hendricks et al. (2023) [30]. The 11 phyla identified across all samples are presented in Figure 2. Twenty-nine classes, 62 orders, 114 families, 312 genera, 602 species, and 876 strains constituted these phyla. Of these strains, 562 were unclassified (de novo) strains and 314 were known, previously published strains.

### 3.3. Sedentary Behavior

Multivariable-adjusted linear regression models used to determine independent associations between sedentary behavior and the alpha diversity and F/B ratio are presented in Table 2. Without adjustment (Model 1), a 1-standard deviation (1-SD) increase in total sedentary behavior (175.2 min/day) was associated with lower observed OTUs (42.6%), Chao 1 Index (41.8%), and Fisher’s Alpha Index (40.4%, *p* < 0.01 for all). When MVPA and BMI were entered into Model 2 and glucose, HDL cholesterol, and triglycerides were added into Model 3, these associations were slightly attenuated but remained significant (*p* < 0.05). There were no associations between sedentary behavior and Shannon Index (*p* > 0.05). Only when the model was adjusted for MVPA, BMI, glucose, HDL cholesterol, and triglycerides (Model 3) was a 1-SD increase in total sedentary behavior associated with a higher F/B ratio (33.1%).

### 3.4. Screen Time

Multivariable-adjusted linear regression models used to determine independent associations between screen time and alpha diversity and F/B ratio are presented in Table 3. Without adjustment, a 1-SD increase in total screen time (194.4 min/day) was associated with lower observed OTUs (34.3%), Shannon Index (32.8%), and Fisher’s Alpha Index (30.1%, *p* < 0.05 for all). The associations for Shannon Index and Fisher’s Alpha Index were attenuated with the addition of MVPA and BMI (model 2, *p* > 0.05). Only the association between screen time and observed OTUs remained significant in the final model. There were no significant associations between screen time and Chao 1 Index or F/B ratio.

## 4. Discussion

The present study evaluated whether sedentary behavior and screen time were associated with human gut microbiota α-diversity and F/B ratio in adults with and without impaired glycemic control. These questions were evaluated by multivariable linear regression analyses. We found that total sedentary behavior was negatively associated with observed OTUs, Chao 1 Index, and Fisher’s Alpha Index. Notably, these associations were independent of relevant covariates including MVPA, BMI, glucose, HDL cholesterol, and triglycerides. This finding supports our hypothesis that sedentary behavior would be inversely associated with α-diversity independent of relevant covariates. Additionally, we found that total screen time was negatively associated with observed OTUs, Shannon Index, and Fisher’s Alpha Index; however, only the association with observed OTUs remained significantly independent with the addition of all covariates. This finding partially supports our hypothesis that screen time would be inversely associated with α-diversity independent of relevant covariates. Finally, total sedentary behavior was associated with F/B ratio independent of MVPA, BMI, glucose, HDL cholesterol, and triglycerides, which supports our hypothesis that sedentary behavior would be positively associated with F/B ratio independent of relevant covariates. Contrary to our hypothesis that screen time would be positively associated with F/B ratio, the associations were not statistically significant in any regression model. These findings suggest that total sedentary behavior and screen time may be associated with compositional changes in human gut microbiota, independent of physical activity, obesity, and risk factors for chronic disease.

To the best of our knowledge, the present study is the first to investigate sedentary behavior and gut microbiota diversity and composition. Bressa et al. (2017), Castellanos et al. (2020), and Zhong et al. (2021) reported differences in gut microbiota between active and sedentary adults; however, they defined sedentary participants as those who do not meet the minimum physical activity guidelines, rather than quantifying their volume of sedentary time [18,19,20]. Because physical inactivity is not synonymous with sedentary behavior—sedentary behavior has different determinants and health consequences than physical inactivity [33]—our study was novel in using validated questionnaires to quantify domain-specific sedentary behavior. Clarke et al. (2014) reported that male rugby athletes had a greater diversity of gut microorganisms (Shannon Index, Chao 1 Index, and observed OTUs) compared to inactive male controls, and concluded that exercise increases microbial alpha diversity in men [15]. Similarly, Castellanos et al. (2020) reported that physically inactive individuals had reduced microbiota richness, as measured by Chao 1 Index, Shannon Index, and observed OTUs, in healthy adults 18–40 years old [19]. Our study extends these findings by specifically quantifying sedentary behaviors and including both men and women across a greater age range and health status.

In the present study, total sedentary behavior was positively associated with F/B ratio, which is similar to the inferences made by Bressa et al. (2017) [18]. Bressa et al. reported a non-significant trend of a lower F/B ratio in physically active women compared to physically inactive women [18], and Larsen et al. (2010) reported that F/B ratio was positively correlated with plasma glucose concentration in men with and without T2D [10]. Additionally, Zhang et al. (2013) reported a negative correlation between insulin resistance and Chao 1 Index [12]. Taken together, these findings indicate that gut microbiome diversity and composition may be a potential mechanism that links sedentary behavior and screen time to risk of T2D; however, additional research in larger, more diverse samples is needed to confirm these findings.

We found that approximately 60% of our participants’ total sedentary behavior time, derived from the SBQ, was spent in leisure time sedentary activities, which includes screen time (e.g., TV viewing). This was a similar proportion to the leisure time sedentary behavior reported by Rosenberg et al. (2010) [23]. Leisure time sedentary behavior was negatively associated with observed OTUs (β = −0.423, *p* < 0.005), Chao 1 Index (β = −0.442, *p* < 0.005), and Fisher’s Alpha Index (β = −0.399 *p* < 0.01). The associations with observed OTUs and Chao 1 Index were independent of MVPA and BMI (β = −0.342 and β = −0.333, *p* < 0.05, respectively), suggesting that the negative association between total sedentary behavior and gut microbiota alpha diversity may be, in part, driven by total leisure time sedentary behavior. The other domains of sedentary behavior from the SBQ—occupational and passive transportation—were not associated with markers of α-diversity.

Our findings from the STQ indicate that although screen time was independently associated with observed OTUs, the associations with other markers of α-diversity were not consistent or independent of relevant covariates. These data are somewhat similar to those reported by Whisner et al. (2018) and Jasbi et al. (2022) in college students [21,22]. Whisner et al. reported no differences in species richness or diversity between quartiles of screen time [22], and Jasbi et al. reported no difference in diversity metrics between high and low screen time, but did find differences in microbiome composition [21]. However, both studies had a few fundamental methodological differences that may contribute to the inconsistencies in findings with the present study. Participants in Whisner et al. and Jasbi et al.’s studies were college students living in residence halls, and their self-reported screen times were grouped into quartiles of high and low, respectively, and evaluated for group differences in microbiome diversity and composition [21,22]. Participants in the present study reported spending over 3.5 h more time per day using a screen than Whisner et al. and Jasbi et al.; however, the questionnaire used in their studies only accounts for leisure time screen use. Additionally, neither study differentiated between types of screened devices being used, which may be an area for future study given the rising prevalence of access to screened devices.

The strengths of our study include the measurement of context-specific sedentary behavior and device-specific screen use via questionnaire. However, several limitations should be considered in interpreting our findings. Our convenience sample was from a small, demographically homogenous community, which limits the applicability of our findings to more diverse population groups. A small sample size of *n* = 47 limits the power of our analyses to detect effects smaller than f^2^ = 0.433. Although age was not significantly associated with our dependent variables, our metabolically healthy participants were an average of 10 years younger than our participants with prediabetes and T2D. We used self-reported sedentary behavior and physical activity, which may be less accurate than objective measurement using accelerometry due to subject recall bias. Finally, our quantification of occupational sedentary behavior and sedentary transportation did not account for the employment status of our participants, which may lead to the underestimation of those variables given that many of our participants were retired.

## 5. Conclusions

In summary, human gut microbiota diversity and composition were negatively associated with sedentary behavior, independent of MVPA, BMI, glucose, HDL cholesterol, and triglycerides. Screen time was negatively associated with one marker of gut microbiota diversity, observed OTUs. These findings suggest that total sedentary behavior, driven by leisure time sedentary activities, and screen time may be a significant influencer of compositional changes in human gut microbiota, which might be a potential mechanism that links sedentary behavior and screen time to risk of T2D.

## Figures and Tables

**Table 1:** Participant characteristics (*n* = 47).

Variable	Central Tendency
Women ^a^	29 (61.7)
Menstrual status ^a^	
Premenopausal	7 (24.1)
Perimenopausal	1 (3.4)
Menopausal	1 (3.4)
Postmenopausal	20 (69.0)
Race/Ethnicity ^a^	
Non-Hispanic White	37 (78.7)
Hispanic	3 (6.4)
African-American/Black	1 (2.1)
Asian/Pacific Islander	4 (8.5)
Other	2 (4.3)
Diabetes status ^a^	
Healthy	29 (61.7)
Prediabetes	8 (17.0)
Type 2 diabetes	10 (21.3)
Risk Factors	
Age (years) ^b^	51.0 (16.1)
BMI (kg/m^2^) ^b^	29.6 (7.2)
Family history of type 2 diabetes ^a^	22 (46.8)
Current smoker ^a^	3 (6.4)
HbA1c (%) ^c^	5.5 (0.8)
Glucose (mg/dL) ^c^	97 (14)
Total cholesterol (mg/dL) ^b^	188.8 (37.9)
LDL cholesterol (mg/dL) ^d^	100.5 (30.1)
HDL cholesterol (mg/dL) ^b^	59.2 (19.2)
Triglycerides (mg/dL) ^c^	117 (77)
Physical Activity and Sedentary Behaviors	
MVPA (MET·min/wk) ^c^	4020 (5541)
Total sedentary behavior (min/day) ^b^	466.3 (175.2)
Sedentary leisure time (min/day) ^c^	231 (191)
Occupational sedentary behavior (min/day) ^b^	143.0 (108.2)
Sedentary transportation (min/day) ^c^	39 (39)
Total screen time (min/day) ^b^	418.9 (194.4)
Television screen time (min/day) ^b^	82.0 (92.2)
TV-connected device screen time (min/day) ^c^	0 (77)
Computer screen time (min/day) ^b^	195.3 (157.0)
Smartphone screen time (min/day) ^b^	84.9 (60.3)
Tablet screen time (min/day) ^c^	0 (0)
Gut Microbiome Alpha Diversity	
Observed OTUs ^b^	169.0 (30.6)
Shannon Index ^b^	3.2 (0.5)
Chao 1 Index ^b^	213.9 (44.6)
Fisher’s Alpha Index ^b^	27.6 (5.3)
F/B ratio ^a^	3.1 (5.6)

Abbreviations: BMI, body mass index; HbA1c, glycated hemoglobin; LDL, low-density lipoprotein; HDL, high-density lipoprotein; MVPA, moderate–vigorous physical activity; OTU, operational taxonomic unit; F/B, *Firmicutes*/*Bacteroidetes*. Note: ^a^, data presented as frequency (percent of sample); ^b^, data presented as mean (standard deviation); ^c^, data presented as median (interquartile range); ^d^, *n* = 41.

**Table 2:** Multivariable linear regression analyses on the associations between total sedentary behavior and indices of alpha diversity and F/B ratio.

	Total Sedentary Behavior		
Model	B [95% CI]	St β	*p*
Observed OTUs (1 SD = 30.6)			
1	−0.080 [−0.131, −0.029]	−0.426	0.003
2	−0.068 [−0.123, −0.012]	−0.360	0.018
3	−0.063 [−0.117, −0.009]	−0.335	0.024
Sq Shannon Index (1 SD = 0.5)			
1	−0.004 [−0.009, 0.001]	−0.241	0.102
2	−0.003 [−0.008, 0.003]	−0.146	0.355
3	−0.002 [−0.007, 0.003]	−0.118	0.416
Chao 1 Index (1 SD = 44.6)			
1	−0.114 [−0.189, −0.040]	−0.418	0.003
2	−0.092 [−0.172, −0.012]	−0.334	0.026
3	−0.090 [−0.170, −0.010]	−0.329	0.028
Fisher’s Alpha Index (1 SD = 5.3)			
1	−0.013 [−0.022, −0.004]	−0.404	0.005
2	−0.010 [−0.020, −0.001]	−0.322	0.034
3	−0.010 [−0.019, −0.001]	−0.314	0.028
Ln F/B ratio (1 SD = 96)			
1	0.002 [0.000, 0.005]	0.284	0.053
2	0.002 [0.000, 0.005]	0.309	0.059
3	0.003 [0.000, 0.005]	0.331	0.044

Abbreviations: B, slope; CI, confidence interval; St β, standardized beta; OTU, operational taxonomic unit; Sq, squared transformed; Ln, natural log transformed; F/B, *Firmicutes*/*Bacteroidetes*. Note: Model 1, unadjusted; Model 2, Model 1 + moderate-to-vigorous physical activity and body mass index; Model 3, Model 2 + glucose, high-density lipoprotein cholesterol, and triglycerides.

**Table 3:** Multivariable linear regression analyses on the associations between total screen time and indices of alpha diversity and F/B ratio.

	Total Screen Time		
Model	B [95% CI]	St β	*p*
Observed OTUs (1 SD = 30.6)			
1	−0.056 [−0.103, −0.010]	−0.343	0.018
2	−0.054 [−0.104, −0.004]	−0.331	0.034
3	−0.059 [−0.111, −0.007]	−0.359	0.026
Sq Shannon Diversity (1 SD = 0.5)			
1	−0.005 [−0.010, −0.001]	−0.328	0.024
2	−0.004 [−0.009, 0.001]	−0.240	0.131
3	−0.002 [−0.007, 0.003]	−0.136	0.389
Chao 1 Index (1 SD = 44.6)			
1	−0.064 [−0.134, 0.005]	−0.269	0.068
2	−0.059 [−0.132, 0.014]	−0.245	0.112
3	−0.067 [−0.145, 0.011]	−0.281	0.089
Fisher’s Alpha Index (1 SD = 5.3)			
1	−0.009 [−0.017, 0.000]	−0.301	0.040
2	−0.008 [−0.017, 0.001]	−0.279	0.072
3	−0.008 [−0.017, −0.001]	−0.278	0.076
Ln F/B ratio (1 SD = 96)			
1	0.001 [−0.001, 0.003]	0.181	0.224
2	0.001 [−0.001, 0.004]	0.195	0.246
3	0.002 [−0.001, 0.004]	0.280	0.121

Abbreviations: B, slope; CI, confidence interval; St β, standardized beta; OTU, operational taxonomic unit; Sq, squared transformed; Ln, natural log transformed; F/B, *Firmicutes*/*Bacteroidetes*. Note: Model 1, unadjusted; Model 2, Model 1 + moderate-to-vigorous physical activity and body mass index; Model 3, Model 2 + glucose, high-density lipoprotein cholesterol, and triglycerides.

## Data Availability

The data presented in this study are openly available under BioProject accession number PRJNA930056. The SRA accession numbers for the reads are SAMN32982567 to SAMN32982614.
